# Treatment of Morgellons disease with doxycycline

**DOI:** 10.1002/ccr3.5148

**Published:** 2021-12-04

**Authors:** Jeff F. Zhang, Keerthy Gopalakrishnan, Daniel J. Molloy

**Affiliations:** ^1^ Jacobs School of Medicine and Biomedical Sciences University at Buffalo Buffalo New York USA; ^2^ SRM Medical College Hospital and Research Centre Kattankulathur Kanchipuram India; ^3^ St. Vincent Health Center, Sisters of Charity Hospital Buffalo New York USA

**Keywords:** Borrelia burgdorferi, dermatopathology, Doxycycline, Lyme disease, Morgellons disease, spirochetes

## Abstract

Morgellons disease (MD) is a rare dermatopathy characterized by nonspecific symptoms and the production of multicolored fibers and granular tissue from diffuse skin ulcerations which are described as being either pruritic or painful. The etiology of MD is currently unknown; previous studies have suggested both psychiatric and infectious causes, with increasing interest over the previous decade in elaborating a possible pathogenesis for the disease secondary to infection by Borrelia species. We report a middle‐aged Caucasian female who developed symptoms of MD in the days following exposure to a tick bite after spending an afternoon hiking through a wooded area. She was subsequently treated with a course of Doxycycline and found on two‐week follow‐up to have complete remission of her symptoms. This case report further supports the theory for an infectious etiology of MD and encourages future studies into its pathophysiology.

## Graphical abstract

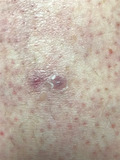

## 1 INTRODUCTION

Morgellons disease (MD) is a skin condition characterized by the abnormal formation of fibers and debris either embedded within or erupting from skin lesions that have been described variably as itchy, sore, stinging, or burning in sensation. Other associated symptoms may include fatigue, joint and muscle pain, cognitive dysfunction, or memory loss.^1^ The condition classically presents in middle‐aged women of Caucasian descent, frequently those with underlying psychiatric disorders. For that reason, MD’s position in the medical literature has been controversial, with practitioners split between classifying MD as a mental disorder related to delusions of infestation, or a true dermatological disease with an infectious etiology. Clinical and histopathological studies in recent years have increasingly found an association between Morgellons disease and spirochetal infection,^2^ with many studies implicating Borrelia species infection in particular.^3^ In this case report, we present a middle‐aged female who developed Morgellons disease days after tick exposure and was successfully treated with a two‐week course of Doxycycline, a regimen with proven efficacy against tickborne Borrelia species.

## 2 CASE PRESENTATION

A 47‐year old Caucasian female with past medical history of anxiety, bipolar disorder, major depressive disorder, and opioid use presented to the emergency department with complaints of a two day history of diffuse, circular skin ulcerations which she described as being both pruritic and painful. She denied any cough, fever, chills, diaphoresis, nausea, vomiting, abdominal pain, dysarthria, facial palsy, or sick contacts in the previous few days. On examination, the patient was found to be hemodynamically stable and in no acute distress. She noted no change in eating habits or recent travel and denied any history of syphilis or Lyme disease. Her home medication use included Suboxone, clonazepam, risperidone, lithium, and an albuterol inhaler. She described having recently spent an afternoon in a wooded area near the river where “there were lots of deer around” and that she had lain in the “tall grass” for hours on a blanket with her boyfriend. At the time of hospitalization, the patient believed she was experiencing either an allergic reaction or multiple insect bites, and she was discharged home with Benadryl and an over‐the‐counter steroidal cream.

**FIGURE 1:**
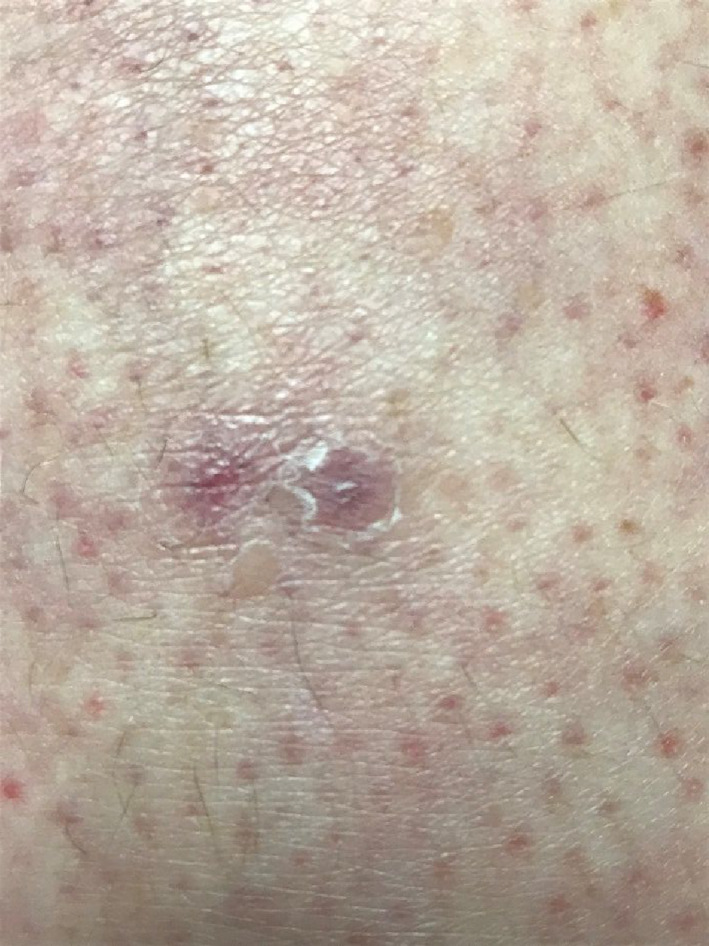
Eruptive lesion with embedded fiber from patient's right thigh

**FIGURE 2:**
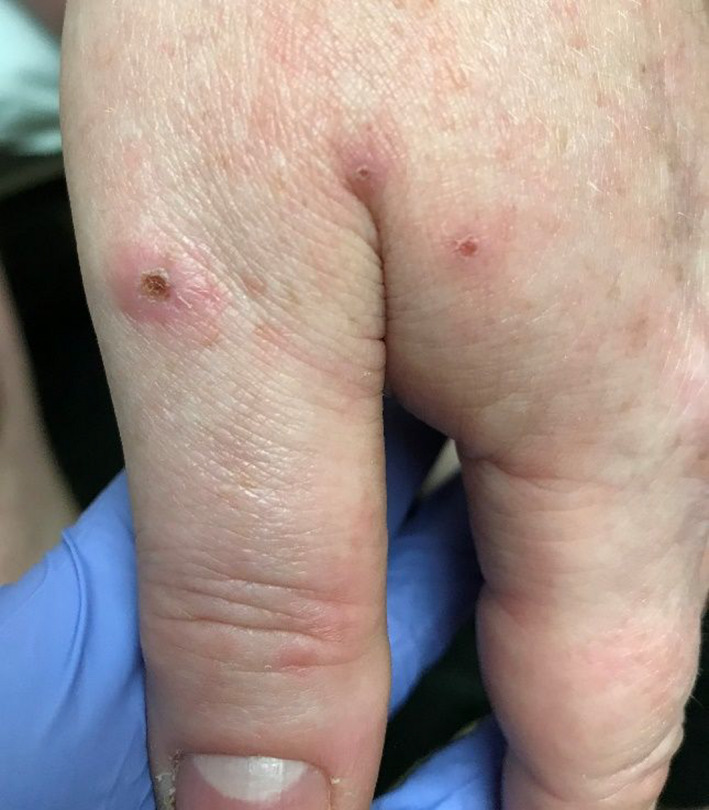
Ulcerated lesions on patient's left hand

**FIGURE 3:**
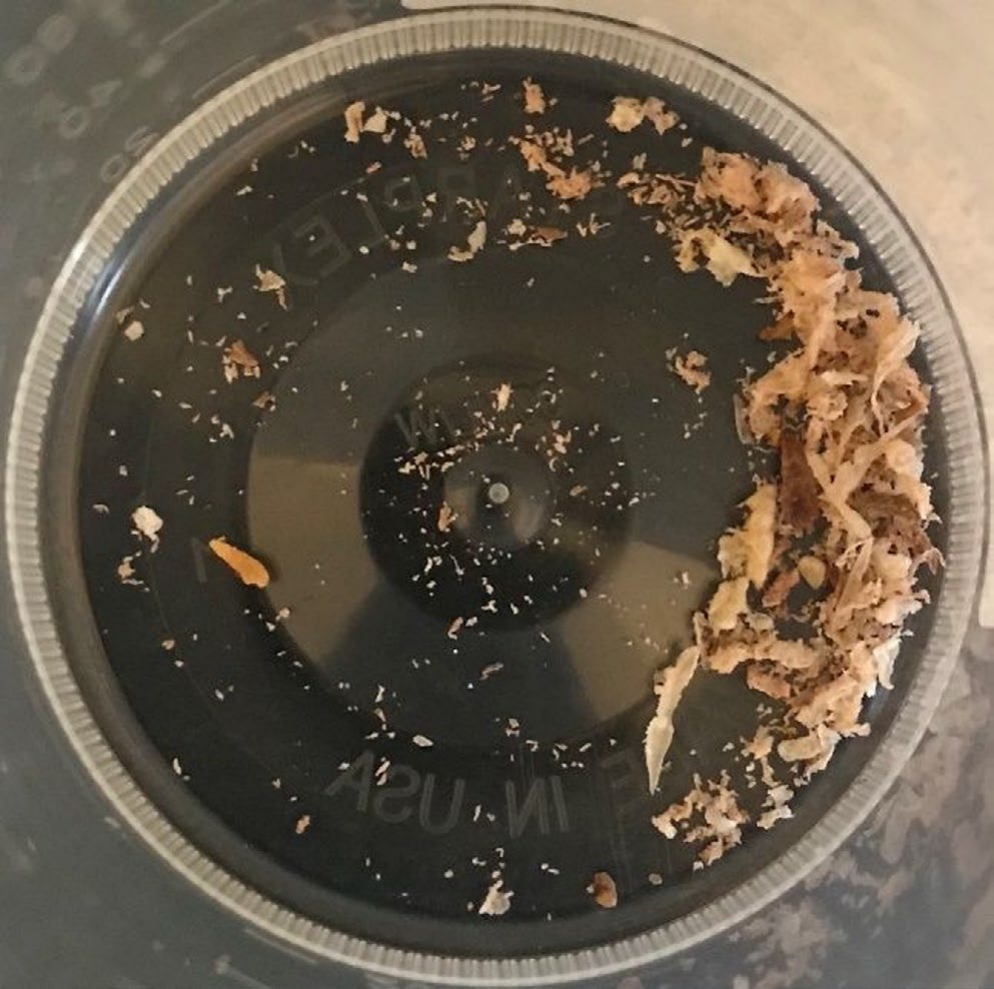
Skin scrapings revealing fibers and debris collected from various lesions

Three days after her initial ED visit, the patient presented to her primary care physician in an acutely anxious state. She complained of a worsening rash and that “worms were coming out of my body.” Images of the patient's skin lesions are shown in Figures 1 and 2. Excoriations and “digging in” the lesions were also noted on physical examination. The patient reported that at some point in the preceding days, a black, circular insect had “crawled out of” or detached from one of her lesions, but she had “flicked it away” rather than capture it. On further questioning, she was able to identify the insect as a tick when presented with a series of pictures of common insects. Topical triamcinolone was prescribed, and recommendations were made to the patient to call an exterminator; however, the patient denied any history of bedbugs or any other home infestation. Repeated follow‐up appointments over the following two months were made, resulting in additional prescriptions for Cetirizine and Loratadine—neither of which relieved her symptoms. She continued to report brownish, granular debris, and multicolored fibers (black, red, and clear) erupting from the lesions (Figure 3), which were consistently described as being pruritic and sometimes “burning” or “stinging” in quality, along with associated symptoms of increased fatigue, difficulty concentrating, and arthralgia over the past few weeks. She remarked that copious amounts of “lint” could be found throughout her house due to the volume of debris and fibers being shed from her lesions. She also reported “spitting stuff out” from lesions in her mouth, though no oral lesions were ever found on physical examination.

**FIGURE 4:**
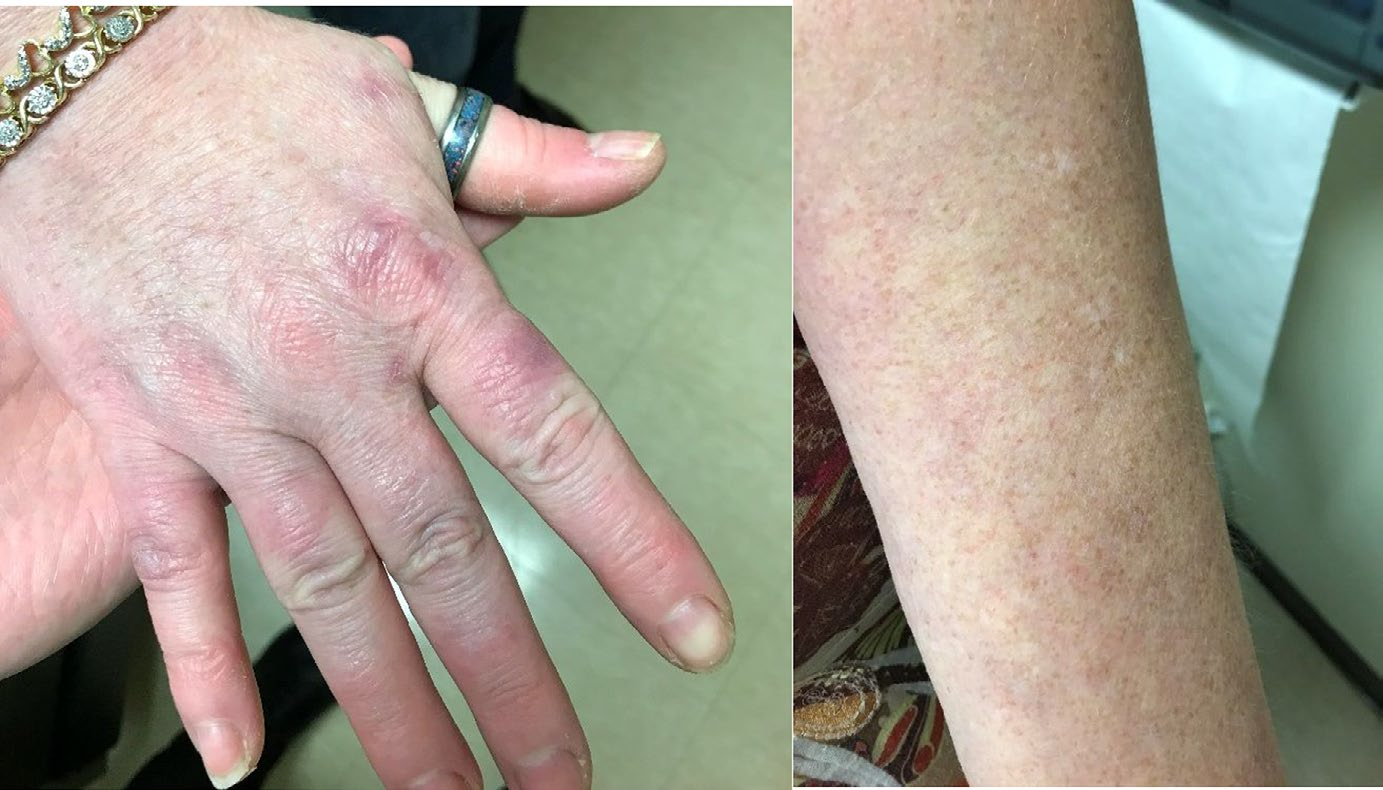
Well‐healed lesions after a fourteen‐day course of Doxycycline

The patient was referred to dermatology, who then prescribed her a two‐week course of topical Clindamycin and Mupirocin creams. She reported some improvement of her symptoms, but multiple lesions were still noted on her subsequent primary follow‐up appointment despite completion of the topical antibiotic therapy. The patient was then started on a fourteen‐day course of oral 100mg Doxycycline BID. At two‐week follow‐up, the patient was found to have significant clinical improvement of her symptoms (Figure 4), with no new ulcerations reported and complete healing of her previously existing lesions. The patient reported good compliance with both the Doxycycline regimen and in continuing her home risperidone and lithium. Lyme serology obtained on day eight of her Doxycycline course was found to be negative for Borrelia burgdorferi IgM and IgG antibodies. CMP and CBC obtained at two‐week follow‐up were within normal ranges. The patient's serum lithium level was found to be low but there were no new complaints of manic symptoms related to her long‐standing bipolar I disorder.

## 3 DISCUSSION

Morgellons disease is a rare and poorly understood skin condition of unclear etiology. A case series from 2012 conducted by the Centers for Disease Control and Prevention that studied 115 patients estimated a prevalence of 3.65 cases per 100,000 individuals in the United States, with a median age of 52 years and both female (77%) and Caucasian (77%) predominance.^4^ While the condition has gained considerable public attention over the past decade, clinical classification of the disease remains split between characterizing MD as a true dermatopathy of physiological origin or simply a manifestation of delusional infestation (also known as Ekbom Syndrome or delusional parasitosis). Efforts to discriminate between these two possible etiologies in various studies have been frustrated by the frequent comorbidity of MD and preexisting psychiatric disorders, with some studies showing the coincidence to be as high as 82%.^5^ Proponents of a delusional etiology for MD point to the established literature for delusional infestation, which presents in an identical demographic and with similar symptoms of persistent itching, biting, and stinging sensations along with widespread, self‐inflicted lesions resulting from the patient attempting to extract the purported parasites from their skin.^6^ In addition, clinical studies have shown success in treating MD using antipsychotic medications in small‐scale trials. A study using Trifluoperazine showed 63% of patients (15 patients from a sample size of 24) in partial or full remission of their symptoms after 6.6 months of therapy.^7^ Other antipsychotics such as olanzapine,^8^ risperidone,^1^ and pimozide^9^ (and even hypnotherapy^10^) have similarly proven effective in relieving symptoms of pruritus and anxiety, resulting in subsequent skin healing following cessation of secondary picking and excoriating behavior.

On the other hand, a number of studies have emerged in the past few years to suggest an association between the development of MD and infection by Borrelia species. A large‐scale study of 1000 patients with Lyme disease identified 60 patients (6%) with symptoms of MD^2^—a coincidence rate significantly higher than prevalence estimates previously reported by the 2012 CDC case series. Comparative animal studies of bovine digital dermatitis, a spirochete‐mediated infection of cattle and sheep which results in similar symptoms of dermatitis and papillomatous lesions with abnormal keratin filament production, further suggests an infectious etiology for MD in humans.^11^ Histological studies have conclusively demonstrated that fibers removed from the skin lesions of patients with MD are composed of keratin and collagen,^12^ not textile fibers as would be expected from patients presenting with a factitious etiology. Both tissue cultures and DNA analysis of skin samples have additionally detected the presence of Borrelia species in the lesions of MD patients.^12^ Studies of ceftriaxone‐resistant cases of Lyme disease in mouse models demonstrated the capacity for Borrelia burgdorferi to invade fibroblasts and keratinocytes.^13^ These findings suggest that genetic or enzymatic dysregulation secondary to cellular infection may be a possible mechanism for the abnormal keratin and collagen formation productive of the characteristic fibers seen in MD, although the particular cellular signaling pathways affected by this pathology have not yet been identified.^13^

At this time, no definitive clinical studies have been performed to determine a proper antibiotic treatment course to treat an infectious cause of MD, though multiple papers have pointed to the need for this future direction of study.^2^, ^3^, ^5^, ^12^ Our approach in treating the patient presented in this case report was to assume an infectious cause for her symptoms after failure of her antipsychotic medications and mild improvement noted with topical antibiotic use in alleviating her symptoms. A case series by Savely and Stricker in 2010 found that 96.8% (118 out of 122 patients) of their included MD patients either tested positive for Borrelia burgdorferi antigens on Western blot or were determined to be clinically positive for Lyme disease by meeting 5/7 diagnostic criteria.^14^ A fourteen‐day course of oral 100mg Doxycycline BID was chosen as the antibiotic regimen for our patient due to its proven efficacy against tickborne Borrelia species, adopted from the 2021 CDC recommendations for the treatment of cutaneous Lyme disease.^15^ Lyme IgM and IgG serology results were obtained at the midpoint of our patient's Doxycycline treatment course and were notably found to be negative. Savely and Stricker noted that at least 18% of their MD patients were found to also be infected with at least one other tickborne organisms, including Babesia microti, Babesia duncani, Anaplasma phagocytophilum, Ehrlichia chaffeensis, and Bartonella henselae.^15^ Other studies of MD patients have detected an onset of symptoms with simultaneous infection by less common Borrelia species, including B. miyamotoi^16^ and B. garinii,^17^ for which a first‐line Lyme disease serology test would not be sensitive enough to detect their presence.^18^

In our case, the patient reported rapid improvement of her symptoms after one week of Doxycycline therapy and complete clearing of lesions at the end of her two‐week course. She remains compliant with her antipsychotic medications, which had not been changed in either dosing or frequency in the past three months. We report this case to bring further attention to the possibility of an infectious etiology for Morgellons disease and its successful treatment with antibiotic therapy.

## 4 CONCLUSIONS

Despite increasing public and clinical interest over the past decade, Morgellons disease remains a mysterious condition with conflicting lines of evidence supporting both psychiatric and infectious etiologies for the disease. This case report suggests an infectious, tickborne agent has a role in its pathogenesis and that Doxycycline therapy can be used to successfully treat the dermatological symptoms of MD.

## CONFLICT OF INTEREST

The authors of this manuscript declare no conflicts of interest involving any of the materials or findings related to this research project.

## AUTHOR CONTRIBUTIONS

Author 1: Jeff Zhang BS: Project conception, data collection, drafting the manuscript, and revising the manuscript. Author 2: Keerthy Gopalakrishnan MD: Project conception and revising the manuscript. Author 3: Daniel J Molloy MD: Project conception.

## CONSENT

Patient consent was obtained for all data collected for this study with written informed consent obtained from the patient to publish this report in accordance with the journal's patient consent policy.

## Data Availability

All data supporting the findings of this study will be made available from the corresponding author upon reasonable request.
